# Supplementary material for: Splice-Junction-Based Mapping of Alternative Isoforms in the Human Proteome
Source: Cell Rep. Author manuscript; Available in PMC 2020 Jan 15. (PMC6961840; doi:10.1016/j.celrep.2019.11.026)
Supplement: 2 [file NIHMS1546469-supplement-2.zip › DF1/PXD010154/Lung/Lung_45_BCAM_ETQRGPLLPPAAGEGGSVSGLLSAMTTIAAR.pdf]

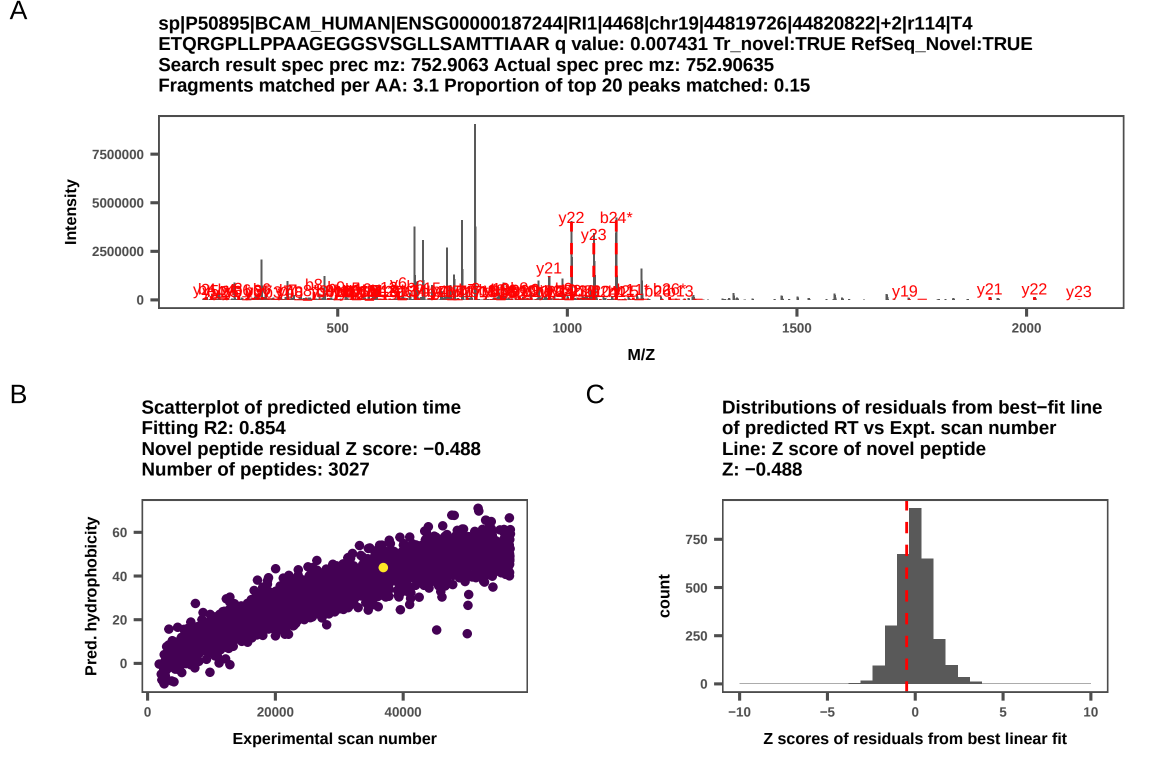
A
sp|P50895|BCAM_HUMAN|ENSG00000187244|RI1|4468|chr19|44819726|44820822|+2|r114|T4
ETQRGPLLPPAAGEGGSVSGLLSAMTTIAAR q value: 0.007431 Tr_novel:TRUE RefSeq_Novel:TRUE
Search result spec prec mz: 752.9063 Actual spec prec mz: 752.90635
Fragments matched per AA: 3.1 Proportion of top 20 peaks matched: 0.15
Intensity
7500000
5000000
2500000
0
y22
b24*
y23
y21
y19
y21
y22
y23
500
1000
1500
2000
M/Z
B
Scatterplot of predicted elution time
Fitting R2: 0.854
Novel peptide residual Z score: −0.488
Number of peptides: 3027
Pred. hydrophobicity
60
40
20
0
0
20000
40000
Experimental scan number
C
Distributions of residuals from best−fit line
of predicted RT vs Expt. scan number
Line: Z score of novel peptide
Z: −0.488
count
750
500
250
0
−10
−5
0
5
10
Z scores of residuals from best linear fit
